# Obstetric care navigation: results of a quality improvement project to provide accompaniment to women for facility-based maternity care in rural Guatemala

**DOI:** 10.1136/bmjqs-2019-009524

**Published:** 2019-11-02

**Authors:** Kirsten Austad, Michel Juarez, Hannah Shryer, Cristina Moratoya, Peter Rohloff

**Affiliations:** 1 Wuqu' Kawoq - Maya Health Alliance, Tecpán, Guatemala; 2 Family Medicine, Boston University School of Medicine, Boston, Massachusetts, USA; 3 Division of Women's Health, Brigham & Women's Hospital, Boston, Massachusetts, USA; 4 Division of Global Health Equity and Social Change, Brigham & Women's Hospital and Children's Hospital, Boston, Massachusetts, USA

**Keywords:** maternal mortality, disrespect and abuse, respectful maternity care, care navigation, quality improvement, Latin America, health disparities, indigenous

## Abstract

**Background:**

Many maternal and perinatal deaths in low-resource settings are preventable. Inadequate access to timely, quality care in maternity facilities drives poor outcomes, especially where women deliver at home with traditional birth attendants (TBA). Yet few solutions exist to support TBA-initiated referrals or address reasons patients frequently refuse facility care, such as disrespectful and abusive treatment. We hypothesised that deploying accompaniers—obstetric care navigators (OCN)—trained to provide integrated patient support would facilitate referrals from TBAs to public hospitals.

**Methods:**

This project built on an existing collaboration with 41 TBAs who serve indigenous Maya villages in Guatemala’s Western Highlands, which provided baseline data for comparison. When TBAs detected pregnancy complications, families were offered OCN referral support. Implementation was guided by bimonthly meetings of the interdisciplinary quality improvement team where the OCN role was iteratively tailored. The primary process outcomes were referral volume, proportion of births receiving facility referral, and referral success rate, which were analysed using statistical process control methods.

**Results:**

Over the 12-month pilot, TBAs attended 847 births. The median referral volume rose from 14 to 27.5, meeting criteria for special cause variation, without a decline in success rate. The proportion of births receiving facility-level care increased from 24±6% to 62±20% after OCN implementation. Hypertensive disorders of pregnancy and prolonged labour were the most common referral indications. The OCN role evolved to include a number of tasks, such as expediting emergency transportation and providing doula-like labour support.

**Conclusions:**

OCN accompaniment increased the proportion of births under TBA care that received facility-level obstetric care. Results from this of obstetric care navigation suggest it is a feasible, patient-centred intervention to improve maternity care.

## Background

Disparities in maternal and neonatal mortality remain one of starkest examples of global health inequality.^1^ Worldwide, 99% of maternal deaths occur in low and middle-income countries (LMIC).^2 3^ Similarly, neonates in the highest neonatal mortality country are 50 times more likely to die in their first month of life than those in the lowest mortality country.^4^ These inequalities are amplified by additional disparities within many LMICs according to income, education and geography.^5 6^ For example, in Guatemala, indigenous Maya women—who represent about half of the female population—are more than twice as likely to die from an obstetric complication compared with non-indigenous mothers.^7^

Failures of health systems to deliver timely, high-quality obstetric care during pregnancy and childbirth drive such disparities.^8–10^ This is especially true in Guatemala, where over half of Maya women deliver at home with traditional birth attendants (TBA) who are themselves indigenous women with little formal education.^11 12^ When complications arise, TBAs are expected to refer women, yet they lack the resources and support to overcome the logistical barriers in the home to public hospital referral chain. Additionally, families often delay or refuse referral when recommended by their TBA due to concerns about the quality of care in public hospitals and fear of disrespectful and abusive care.^13^

To date, most community-level interventions to make home births safer, including our own prior work, have focused on improving detection of obstetric complications.^9 14–17^ However, detection alone will not lower mortality rates unless the disjointed referral pathway between TBAs and hospital providers is also strengthened and women become more receptive to hospital maternity care. These needs align with several of the quality of care domains from the WHO’s Quality of Care Framework for maternal and newborn healthcare, namely functional referral systems and effective communication, respect and dignity, and emotional support.^18^

**Figure 1:**
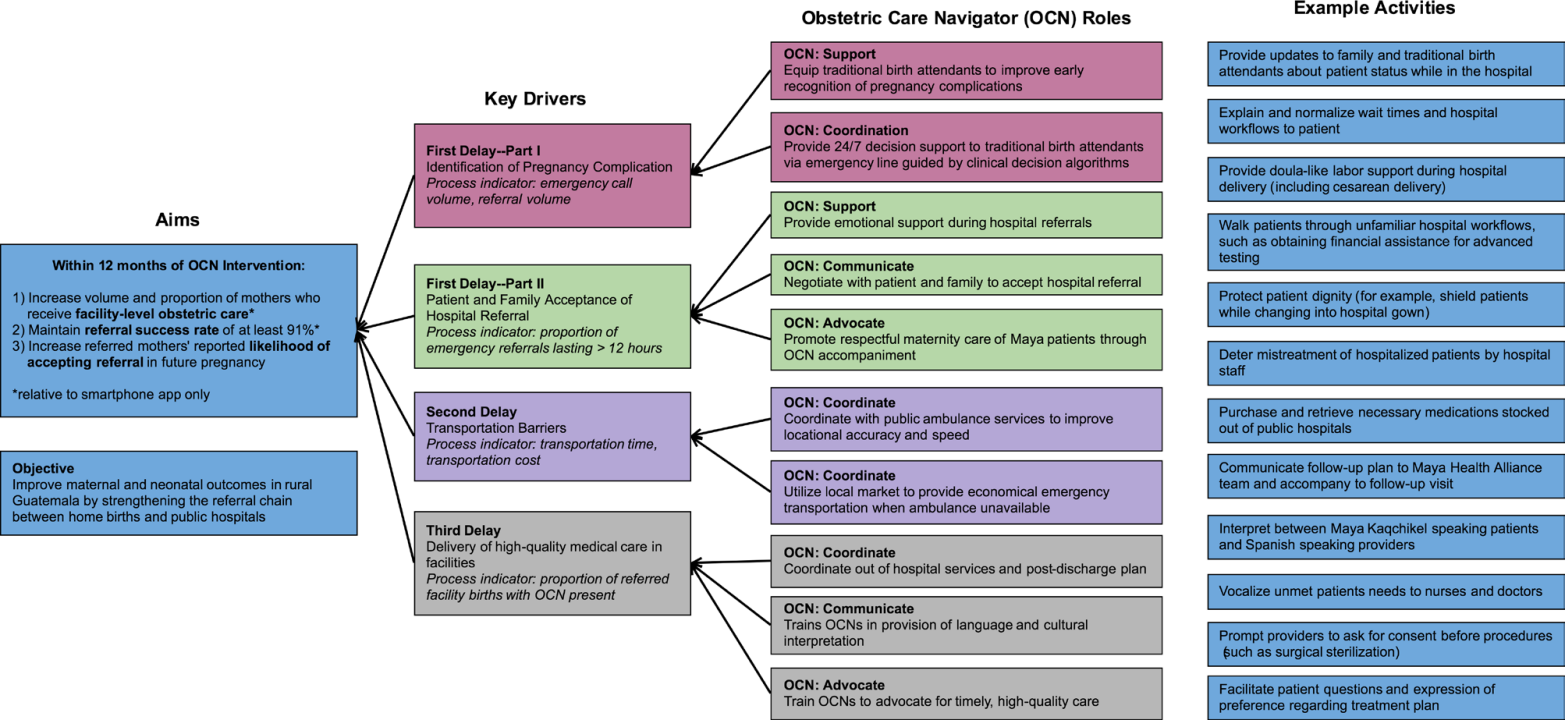
Driver diagram for obstetric care navigator intervention based on Three Delays Model of maternal mortality. Key improvement aims are specified and obstetric care navigator roles and example activities as they relate to the primary drivers of delay in maternal care indicated. OCN, obstetric care navigator.

In this project, we developed an innovative model of obstetric care navigation.^19^ Obstetric care navigators (OCN) are Maya women trained to provide accompaniment and care coordination to mothers. Our OCN model borrows from care navigation and doula labour support models, both evidence-based interventions in high-income settings.^20–22^ OCNs provide a formal linkage between TBAs and hospital-level care and improve the quality of care by coordinating referral logistics, interpreting between non-Spanish-speaking patients and hospital providers, advocating for respectful maternity care, providing emotional and doula-like support, and more (figure 1).

Here we report on the results of this first ever OCN intervention, implemented within an ongoing collaboration with TBAs equipped with mHealth technology to improve their detection of high-risk pregnancies and birth complications.^17 23^ Using a quality improvement (QI) implementation and evaluation approach, the intervention tested the ability of OCNs to improve the referral process from home care to facility-level obstetric care in rural Maya villages in Guatemala.

## Methods

### Context

Maya Health Alliance is a primary care organisation serving rural indigenous Maya communities in Guatemala. Since 2007, Maya Health Alliance has provided technical assistance to TBAs who provide prenatal care to and attend home deliveries of mothers from the municipality of Tecpán (population 95 000) in the department of Chimaltenango, located in the Western highlands of Guatemala.

**Figure 2:**
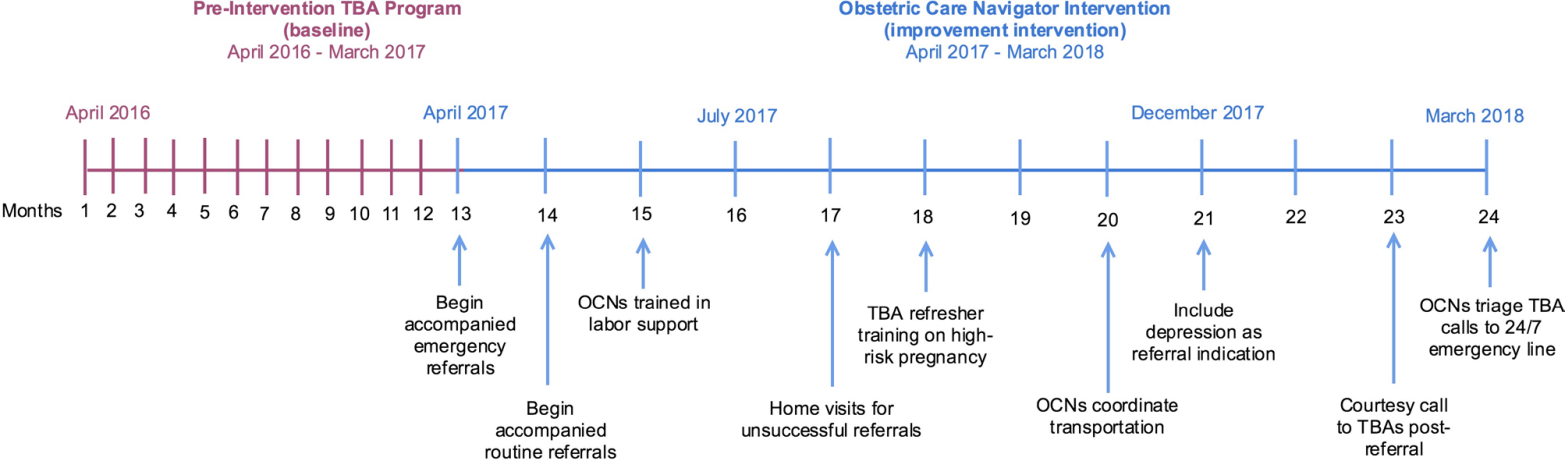
Timeline (in months) of preintervention baseline (traditional birth attending programme, in red) and obstetric care navigator intervention (in blue). Vertical arrows denote relevant changes to the improvement intervention over time. OCN, obstetric care navigator; TBA, traditional birth attendant.

The baseline data for this intervention are a recent 12-month programme in which Maya Health Alliance tested a smartphone application to improve detection of pregnancy complications.^17 23^ In all, 44 TBAs were equipped with the application. If a need for emergency facility referral care was identified, Maya Health Alliance supported mothers by notifying public emergency transportation and offering financial support for out-of-pocket hospital expenses. During the 12-month programme (April 2016 to March 2017; figure 2), 799 births occurred, and the application facilitated a median of 13 emergency referrals per month, with a 91% overall referral success rate. Based on these outcomes, Maya Health Alliance adopted the application-assisted referral model as standard of care.

### OCN design process

While most referrals were successful during this preliminary work, there were limits to the team’s capacity to provide individualised support for a wide range of indications for referral. Our improvement project sought to increase the proportion (and volume) of mothers receiving facility-level care by broadening indications for referral to include more high-risk conditions, such as prolonged labour or need for maternal-fetal medicine consultation due to a pre-existing condition. The goal was to increase these referrals without a reduction in the programme’s overall referral success rate.

To design the intervention, staff and TBAs reviewed the existing literature and personal experiences to identify weaknesses in the referral chain. First, fear of public hospitals led patients and families to refuse or delay referral. Common drivers of fear included mistreatment by hospital staff, inability to communicate with providers (who do not speak Mayan languages), inability of family or TBAs to accompany patients into care areas, perceived low quality of services and out-of-pocket costs.^13^ Second, coordinating logistics with patients and paramedics proved difficult and time consuming. Third, Maya Health Alliance staff had difficulty communicating with providers in public hospitals to explain the indication for patient referral, advocate for care and understand the diagnostic workup and recommended follow-up. We used a key driver diagram to map these observations onto the Three Delays Model, which understands the drivers of maternal mortality as delays in decision to seek, reach or receive adequate facility-level care.^24^

Next, we created the role of the OCN to overcome the three delays. The four key functions of OCNs—coordinate, communicate, support and advocate—correspond directly as shown in the driver diagram (figure 1). We recruited women from local Maya villages who were bilingual, facile with technology and willing to fulfil the intensive call schedule as OCNs. We provided them with hands-on training in medical interpretation, labour support, conflict de-escalation, and other skills describe more fully elsewhere.^19^

### OCN intervention

The OCN intervention was implemented from April 2017 to March 2018 with 41 TBAs (figure 2). During this time, TBAs continued to provide home-based care with the aid of the smartphone application. All patients under the care of TBAs were eligible for accompaniment by an OCN. When TBAs—supported by Maya Health Alliance staff—detected the need for emergency facility-level care, the on-call OCN was notified and coordinated ambulance service for transport. In cases where patients or families refused referral, the OCNs travelled to the patient’s home to evaluate barriers to referral. OCNs also provided accompaniment for routine hospital visits, including scheduled follow-up after an emergency or maternal-fetal medicine consultation for high-risk pregnancies (including diabetes, chronic hypertension, history of pre-eclampsia or gestational hypertension and prior intrauterine fetal demise).

Prior to implementation, project staff met with leadership of each of the four government-run health facilities within the project’s catchment area to finalise the OCN roles and to develop workflows for patient management. Subsequently, information sessions with physicians, nurses and support staff at each facility explained the project, introduced the OCNs and solicited feedback. As a result of this planning, OCNs were allowed to enter all patient care areas, including operating rooms, and remained at the patient’s side until transfer to the postpartum ward or discharge.

To facilitate the intervention, we formed a QI team, which consisted of medical and nursing leadership from Maya Health Alliance as well as OCNs and TBAs. This team met every 2 weeks to review data, identify areas for improvement and rapidly adapt the intervention to improve performance according to the outcome and process measures (figure 1) using a ‘Plan-Do-Study-Act’ methodology.^25 26^ Our primary QI outcomes were referral success rate and proportion of deliveries receiving facility-level care. Secondary outcomes were referral volume and duration, defined as the time from recognition of referral indication to appropriate medical care.

### Data collection

Our project took advantage of existing data collection infrastructure at Maya Health Alliance. As part of the ongoing smartphone TBA project, community health workers (CHW) bilingual in Spanish and Maya Kaqchikel perform home visits for pregnant patients cared for by TBAs (‘TBA cohort’). At this visit, signed informed consent for use of individual-level data was obtained and a brief structured interview captured demographics and obstetric history, which was documented in the electronic health record. Of note, all women under TBA care were eligible for accompanied referral whether or not they had yet been reached for a CHW home interview. Approximately 4–8 weeks after delivery, CHWs conducted a postpartum visit to homes of mothers in TBA cohort to document patient-reported pregnancy outcomes and complications.

Calls to the emergency line from TBAs were also documented in the patient’s electronic health record. OCNs completed a structured referral form after each accompanied referral, which documented patient age, parity, gestational age, indication for facility transfer, duration of referral, referral outcomes and cost. A physician reviewed each chart and documented final clinical diagnoses, which were confirmed by facility chart review when necessary.

TBAs reported both deidentified monthly birth volume, maternal deaths, and neonatal deaths and stillbirths in home deliveries (including those not yet interviewed or accompanied). Online supplementary figure 1 provides a visual depiction of these patient subgroupings. Unaccompanied referrals occurred due to OCN unavailability or family’s preference for referral to non-public hospital.

10.1136/bmjqs-2019-009524.supp1 Supplementary data

### Statistical analysis

Data were abstracted from the electronic record with the help of a computer programmer. We used Stata V.14 (College Station, TX) to generate descriptive statistics and Minitab V.17 (State College, PA) to construct run and control charts.

Patient clinical and demographic characteristics were summarised using median and IQR for continuous variables and raw percentages for categorical variables. Maternal and neonatal death and stillbirth rates were compared between the women who received OCN accompaniment and the remaining TBA cohort using a χ^2^ test. Similarly, occurrence of referral, location and mode of delivery, as well as select clinical outcomes (uterine rupture and hypertensive disorders) were compared between all mothers with postpartum interview and the subset of them who had an accompanied referral. Lastly, indications for referral were grouped in relevant clinical categories and reported as raw percentages.

To assess the impact of the intervention on improvement outcomes, we used statistical process control (SPC) methods.^27^ In SPC the concept of a ‘special cause’—a pattern in data that is unlikely to be due to chance alone—parallels the concept of ‘statistical significance’ in traditional statistical methods. We visualised process data through the construction of run charts, plotting monthly births, phone calls, and emergency and routine referral volume over the project. We used data from the 12 months preceding the intervention (TBAs using the smartphone application but without OCN referral assistance) to construct the baseline. Subsequently, we constructed control charts to examine the proportion of births resulting in emergency or routine facility-level care. We also examined the proportion of referrals that were successful, average time to referral completion and average cost. To determine special cause, we constructed control limits for each control chart (equivalent to ‘critical region’ in a hypothesis test), and applied a conservative special cause rule, requiring that special cause be inferred only for data points lying outside the control limits.

To control for possible autocorrelation, we conducted a sensitivity analysis using interrupted time series analysis for the proportion of births resulting in facility-level care with the ITSA command in STATA.^28^ Newey-West SE estimates were used, and the ACTEST command was used to ensure that the model accounted for the correct autocorrelation structure.^29^

## Results

### Details of the intervention and evolution over time

The improvement intervention lasted from April 2017 to March 2018. The QI team met every 2 weeks to review process outcomes and discuss clinical cases from the OCN referral pool. Major adjustments to OCN roles occurred during the project in response to bimonthly review of project outcomes. For example, transportation was initially coordinated by another team member—due to the time-sensitive nature of emergency referrals—but this task was transferred to OCNs in month 7 once they could efficiently perform other responsibilities. Similarly, prenatal surveys detected numerous women with severe depression. While depression was not initially included as a referral indication, this changed in month 8 when OCNs began facilitating referrals to a nearby psychologist for evaluation and treatment. A timeline of major adjustments is provided in figure 2.

### Characteristics of the patient cohort

Given the difficult rural geography of the service area only 485 (57%) women in the TBA cohort were reached for prenatal interview. As such full demographic data and obstetric history were available for the 467 (55%) women who were reached and gave consent, including 196 women (71%) who received OCN services. Overall women were 27 years old (IQR 22–31) and had two prior deliveries (IQR 1–4) excluding nulliparous women (who comprised 43% of the overall cohort). Among non-nulliparous women, 15.1% had a prior caesarean delivery, and 26% had received facility-level care in their most recent prior pregnancy, with 16% of deliveries attended by a skilled provider. In total, 15.8% had a pre-existing high-risk prenatal condition. The subgroup of 196 patients who received OCN accompaniment was similar to the larger TBA cohort: 27 years old (IQR 20–30.5) with an average two prior deliveries (IQR 1–4), excluding nulliparous women (49.5% of this subset). About one-third (32.8%) had received facility-level care in their most recent prior pregnancy. No statistical difference in demographics was found between the 467 patients and the subset of 196 who received OCN referral.

**Figure 3:**
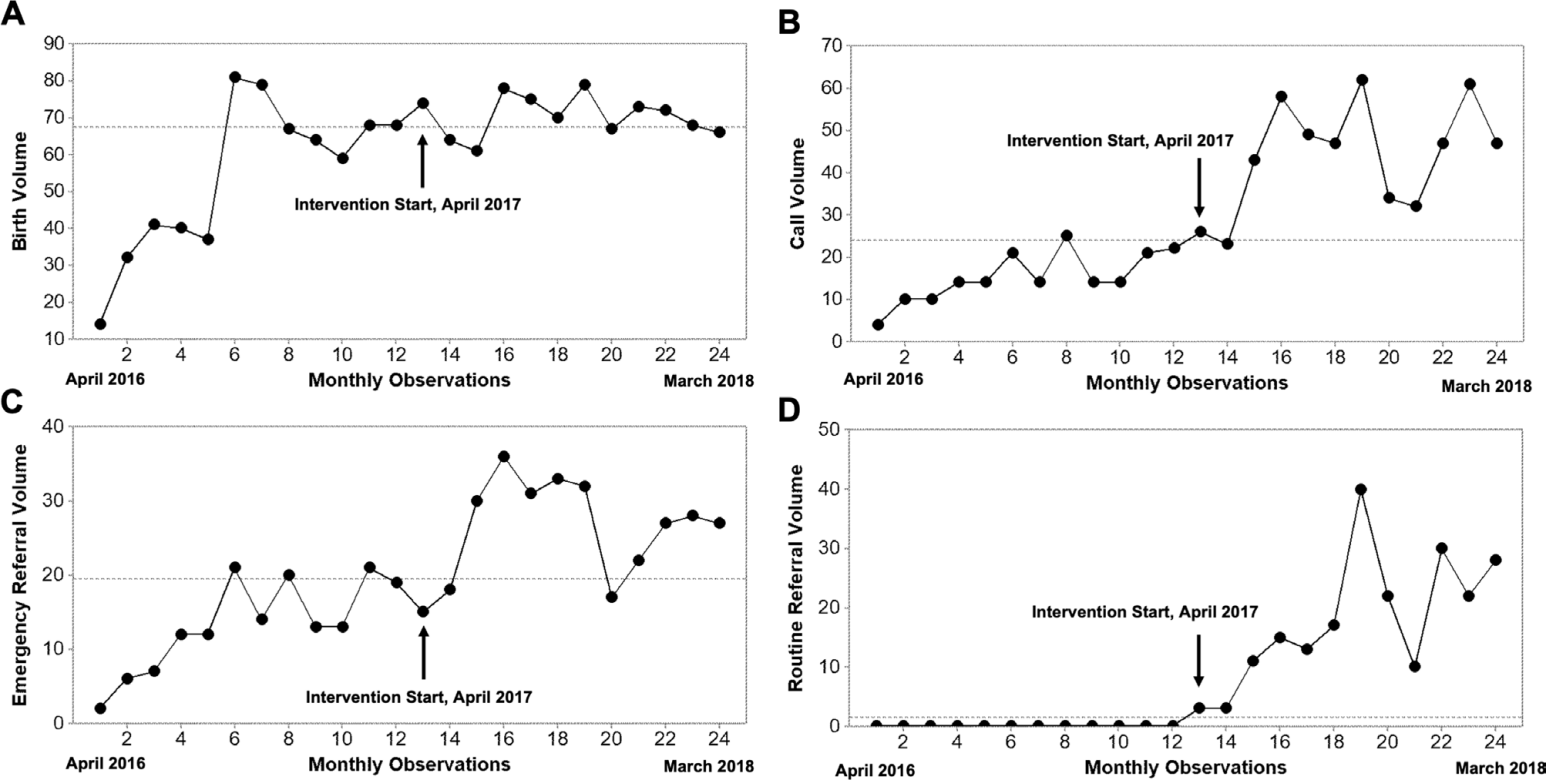
Run charts depicting key process indicators for the obstetric care navigator improvement intervention. (A) Monthly observed birth volume. (B) Monthly volume of calls to the triage phone line from traditional birth attendants (TBA). (C) Monthly volume of emergency obstetric referrals initiated by TBAs. (D) Monthly volume of routine obstetric referrals initiated by TBAs. Indicators are plotted for the preintervention period (months 1–12, April 2016 to March 2017) and the intervention period (months 13–24, April 2017 to March 2018). Dashed horizontal line represents the median for the entire observation period; arrows indicate the start of the obstetric care navigator (OCN) improvement intervention.

A run chart of monthly birth volume is shown in figure 3A. In the baseline period (April 2016 to March 2017, months 1–12) the number of monthly births in the cohort increased steadily as new staff and participating midwives were on-boarded but stabilised during months 6–12, similar to other volume outcomes reported in the following section. The median birth volume during the intervention period (April 2017 to March 2018, months 13–24) was 71 births per month.

### Referral volume and process outcomes

TBAs generated 529 calls to the triage phone line over the 12-month intervention (monthly median 47, IQR 33–53.5). A run chart of call volume is shown in figure 3B. In the preintervention period the median was 14 calls/month, increasing to 47 in the intervention period. Over 12 months, the intervention completed 316 emergency and 214 routine referrals (figure 3C, D). Emergency referral volume increased from a monthly preintervention median of 13 to 27.5 during the intervention, although this increase was most marked in the first few months of the intervention and then declined (figure 3C). Routine referrals did not occur in the preintervention period, but rose to a monthly median of 16 during the intervention (figure 3D). In total, 276 women received OCN services during the intervention period (table 1).

**Table 1:** Outcomes of completed pregnancies receiving obstetric care navigator (OCN) accompaniment during improvement intervention compared with those who did not receive services

Characteristic	OCN accompaniment		No OCN accompaniment		P value
	n	Value (%)	n	Value (%)	
Referral during pregnancy	276/276	100	73/504*	14.5	*<* *0.001*
>1 referral**†**	77/276	27.9	–	–	–
Emergency referral	231/276	83.7	–	–	–
Home delivery‡	73/276	26.5	426/506	84.2	*<* *0.001*
Caesarean delivery‡	97/276	32.6	30/506	6.0	*<* *0.001*
Stillbirth**§**	4/276	1.5	0/571	0	*0.007*
Neonatal death**§**	6/276	2.2	13/571	2.8	0.732
Maternal death**§**	0/276	0	0/571	0	–
Uterine rupture‡	1/276	0.4	0/506	0	0.175
Hypertensive disorders of pregnancy‡	23/276	8.3	13/506	2.6	*<* *0.001*

Italics signify statistical signifiance at p <0.05.

*Data missing for two patients.

†Excludes nulliparous women.

‡These outcomes were collected in postnatal interviews which were conducted with 782 mothers in the traditional birth attendant (TBA) cohort, or 506 of those who did not receive obstetric care navigator (OCN) support.

§These clinical outcomes represent the entire TBA cohort (n=847) as they were reported by TBAs on a deidentified basis and thus did not require postnatal interview or patient consent.

**Figure 4:**
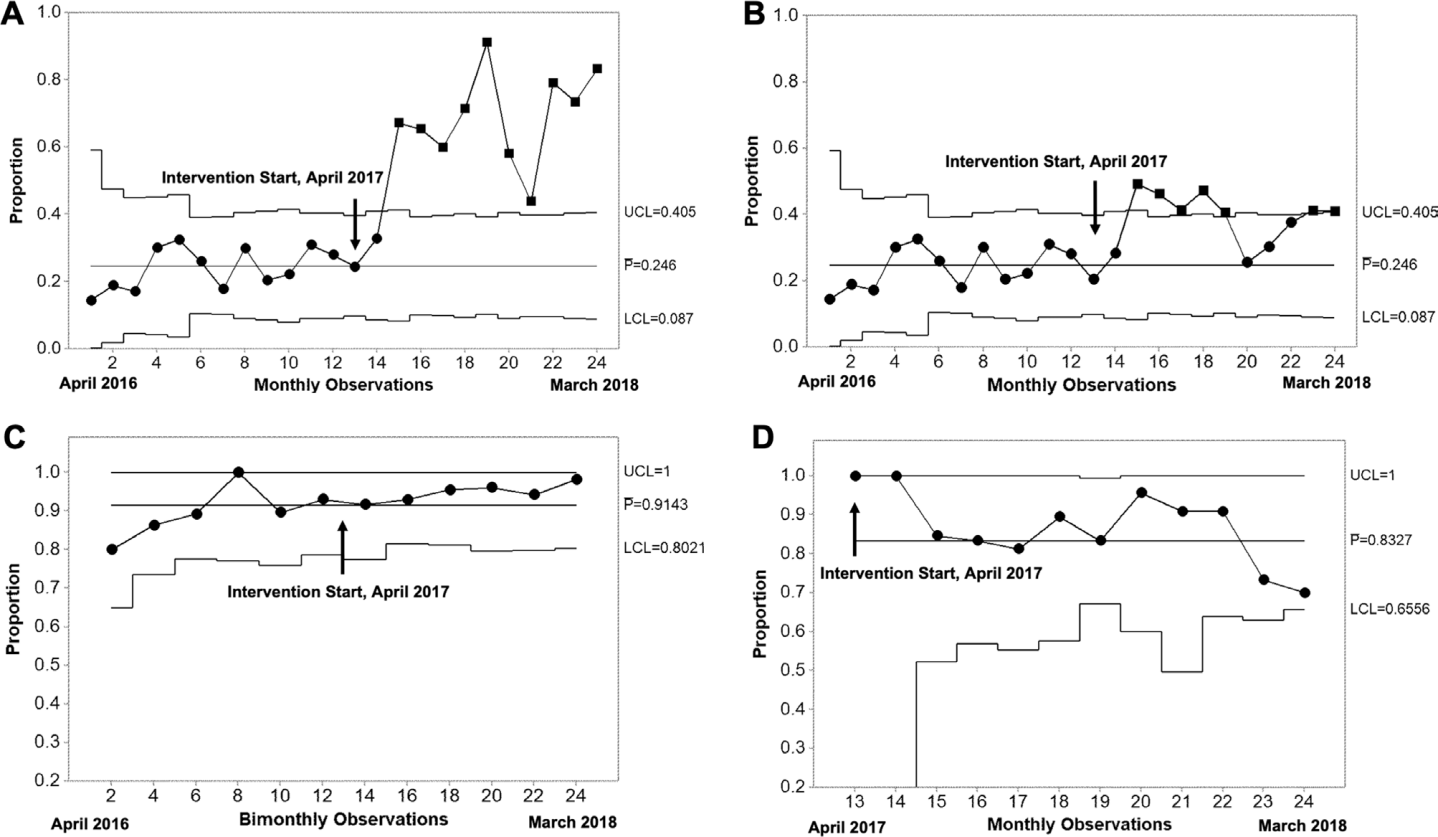
Proportion control charts depicting key process indicators for the obstetric care navigator improvement intervention. (A) Proportion of all monthly births with facility-level care through routine or emergency referral mechanisms. (B) Proportion of all monthly births with facility-level care through emergency referral mechanisms alone. (C) Proportion of emergency referrals that were successfully completed. Given the high success rate of emergency referrals, indicators were grouped bimonthly in order to obtain enough non-conforming units (unsuccessful referrals) to construct the control chart. (D) Proportion of monthly routine referrals that were successfully completed. In each chart, the upper (UCL) and lower control limits (LCL), and the baseline proportion ($\overset{-}{P}$) calculated from the preintervention period are shown, with the exception of (D), where no preintervention data were available and $\overset{-}{P}$ is therefore calculated from the intervention period. Special cause is indicated by squares at the relevant time points. All indicators are plotted for the preintervention period (months 1–12, April 2016 to March 2017) and the intervention period (months 13–24, April 2017 to March 2018). Arrows indicate the start of the obstetric care navigator (OCN) improvement intervention.

In order to assess for shifts in the rate of facility-level care meeting special cause variation from the preintervention to intervention period while controlling for month-to-month variation in births, we constructed control charts depicting the proportion of monthly births receiving facility-level care. Figure 4A shows the total proportion of all deliveries receiving facility-level care (through either emergency or routine mechanisms), demonstrating that special cause was obtained in month 14 and maintained throughout the intervention. Similarly, figure 4B shows the control chart for proportion of emergency referrals alone, again demonstrating early special cause, although with a tendency to decrease over time as emergency referrals were replaced by routine referrals. The mean preintervention proportion of deliveries receiving facility-level care was 24%±6%, increasing to 62%±20% in the intervention period. To investigate if increasing referral volume led to decreased referral success rate over time, we constructed control charts for the proportion of emergency (figure 4C) and routine (figure 4D) referrals successfully completed. No special cause detected for a decrease in referral success rate.

**Figure 5:**
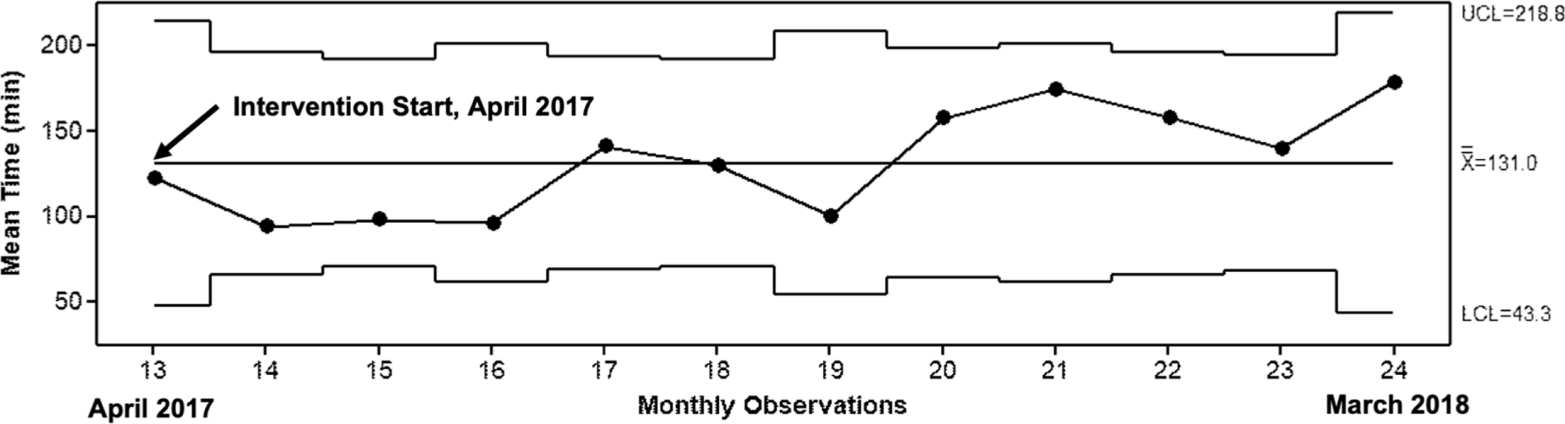
X-bar control chart depicting mean referral time for emergency referrals during the improvement intervention. The upper (UCL) and lower control limits (LCL), and the mean during the intervention period ($\overset{-}{X}$) are shown. An arrow indicates the start of the improvement intervention. The arrows indicate the start of the obstetric care navigator (OCN) improvement intervention.

We constructed X-bar ($\overset{-}{X}$) control charts to examine changes in mean time for emergency referrals and cost per referral. Baseline data from the preintervention period for cost and referral time were not available. The mean emergency referral time was 131±138 min, with no observed change over the intervention (figure 5). The mean cost of referrals was 212.8±170.6 Guatemalan quetzales (approximately US$28), with special cause for higher cost in the first observation period and a subsequent decline in mean, as lower cost routine referrals became more frequent (online supplementary figure 2A). The mean cost of emergency and routine referrals was 304.8±137.9 quetzales (approximately US$40) and 111.4±143.8 quetzales (approximately US$15), respectively. There was no special cause variation in the mean cost of either emergency or routine referrals over the intervention period (online supplementary figure 2B, C).

10.1136/bmjqs-2019-009524.supp2 Supplementary data

### Sensitivity analysis

To control for possible autocorrelation in the data set, we used interrupted time series analysis to examine the proportion of births resulting in facility-level care (online supplementary figure 3). The total proportion of deliveries receiving facility care increased 13.8% (95% CI −7.3 to 35.0) in the first month of the intervention, followed by a 3.5% monthly increase (95% CI 0.8 to 6.2) throughout the intervention period.

10.1136/bmjqs-2019-009524.supp3 Supplementary data

### Patient-level outcomes

Detailed pregnancy outcome data were available for 92% of the TBA cohort who received a postnatal home visit and consented (798 approached, 782 gave consent). This included 276 accompanied mothers and 506 mothers who did not receive OCN services. Table 1 compares outcomes of accompanied and unaccompanied mothers. Higher rates of facility and caesarean delivery, stillbirth and hypertensive disorders of pregnancy were observed in referred patients. No statistical difference was found among other variables.

The most common reasons for TBA-initiated emergency referrals were the following: signs and symptoms of hypertensive disorders of pregnancy (19.6%), prolonged labour (13.2%), haemorrhage (10.8%), premature rupture of membranes (9.6%) and abdominal pain (5.6%). The most common reasons for routine referrals were signs and symptoms of or history of hypertensive disorder (30%), signs of infection (most commonly urinary tract infection; 13.2%), depressive symptoms (12%), abdominal pain (7.2%) and prior caesarean section (4.8%).

To assess the ability of TBAs to accurately identify indications for referral, we calculated the inter-rater reliability of the top five indications for emergency referral with the final medical diagnosis. Overall agreement was 61%, and Krippendorff’s alpha was 0.52, indicating moderate agreement. Agreement was highest for abdominal pain (86%) and prolonged labour (88%), and lower for hypertension (55%), haemorrhage (52%) and premature rupture of membranes (33%). In most cases of disagreement, the final medical diagnosis still remained an important indication for referral. However, 6%, 26% and 17% of hypertension, haemorrhage and premature rupture of membrane referrals, respectively, were ultimately diagnosed as uncomplicated pregnancies.

## Discussion

Barriers to obstetric referral contribute to high rates of maternal morbidity and mortality worldwide, especially among poor, rural and indigenous women. Here we implemented an obstetric care navigation intervention to improve collaboration between TBAs and health facilities in rural Guatemala. We found that implementation of OCN support correlated with an increase in the proportion of pregnancies receiving facility care (figure 4A, B), as compared with the preintervention period during which women received logistical support but not accompaniment (62% vs 24%). Much of this increase was through facilitating non-emergency referrals for pregnancies with high-risk features (figure 3D). Despite an increase in referral volume meeting special cause variation (median monthly referrals increased from 13 to 47) we did not detect any decline in referral success rate (figure 4C, D). The average duration of an emergency referral during the intervention was 131±138 min. Since time to referral completion was not collected during the preintervention period, we could not evaluate for reductions in time delays. While stillbirths were higher among mothers in the OCN intervention (p=0.007), this may be due to misclassification of stillbirths as neonatal deaths by TBAs. We were unable to draw conclusions on the impact of the OCN intervention on maternal mortality given that none occurred in either the TBA cohort.

Overall these findings are promising, given the dearth of interventions which have shown a positive impact on facilitating access to obstetric care.^8 9 14 30^ For example, a recent Cochrane review of community-level interventions found that none of those tested to date significantly impacted maternal morbidities or rates of skilled delivery.^9^ In addition, Guatemala is a particularly challenging environment for obstetric referrals. A recent study of the Global Network for Women and Children’s Health—conducted in the Democratic Republic of the Congo, Pakistan, Kenya, Zambia and Guatemala—tested community-based antenatal ultrasound screening as a strategy to improve pregnancy outcomes through detection and referral.^31^ Guatemala had the lowest rate of referral completion at 52%, compared with more than 90% in the African nations. The study concluded that unsuccessful referrals were driven by transportation hurdles, and by barriers within hospitals themselves. One unique feature of our OCN approach is that it impacts the entire continuum of obstetric care (figure 1), and therefore may help to overcome these complex, multilevel barriers.

While care navigation as a strategy for improving care in LMICs has been evaluated for other medical needs, such as cancer care, to our knowledge this is the first published experience using care navigators for obstetric care.^32^ A strength of the project’s improvement-based design is that it allows us to isolate the effect of accompaniment. To evaluate impact, we used baseline data on referrals from the same group of TBAs whose patients, in the year prior to this intervention, were given financial and logistical assistance for transfer to public hospitals. The increase in rate of facility-level obstetric care we subsequently observed with the implementation of OCN accompaniment emphasises that there are multiple barriers to successfully accessing care in Guatemala beyond financial ones.

A common reason for obstetric care facilitated by OCNs was hypertensive disorders of pregnancy, representing 19.6% and 30% of emergency and routine referral indications, respectively. In addition, the rate of hypertensive disorders among OCN-accompanied women was 8.3% (table 1), compared with a rate of 3.5% in the preintervention period that we previously reported.^17^ These findings represent circumstantial but important evidence that the OCN intervention may increase the detection and management of these conditions. According to a recent study by the Global Network for Women and Children’s Health in the same geographic area of Guatemala, hypertensive disorders represented the strongest predictor of maternal mortality.^33^ Taken together, this means that OCNs have the potential to impact an important driver of maternal outcomes in Guatemala.

Another important quality concern of this intervention is that it may unintentionally increase the proportion of individuals referred to facility care—and increase the risk of caesarean delivery—when unnecessary or for erroneous indications. To evaluate this, we calculated Krippendorff’s alpha for emergency referrals, demonstrating moderate agreement (0.52). However, even in cases without agreement, the majority of final medical diagnoses supported the need for referral. Taken together, these results suggest that, on balance, the intervention appropriately triaged most individuals for necessary care.

Perceptions of the quality of and need for care are important drivers of patient demand for skilled delivery. Previous literature has shown that marginalised women are most susceptible todisrespectful and abusive in childbirth facilities, which contributes to the high rate of home delivery.^34^ The rise in institutional obstetric care rates we observed here suggests that OCNs effectively decreased fear of institutional obstetric care. We attempted to confirm this during our project by surveying patient satisfaction, but efforts were unsuccessful due to poor comprehension of the concept of satisfaction despite multiple rounds of survey revision. This barrier has been reported by others in field and calls attention to the need for more research to develop tools for measuring satisfaction and perceptions of quality, especially among rural and low-literacy populations.^35^ It also raises the important question of whether satisfaction is the best desired outcome measure, given that low baseline expectations of care may cause positive reporting of satisfaction even when objective quality is low.^36^

It is important to highlight the limitations of our findings. First, because only half of the TBA cohort was reached prenatally, it is possible that systematic differences in unreached patients were not detected and, therefore, weakenis our conclusion that mothers who received OCN accompaniment are similar to the remaining TBA cohort according to basic demographics (such as age and parity). Similarly, pregnancy complications in the patients who did not receive OCN accompaniment were reported by patients and not corroborated by chart review. Second, as an improvement intervention, our study lacks a rigorous control, so we cannot rule out that the increase in facility-level obstetric care we attribute to OCNs was in fact influenced by other unaccounted factors, such as ongoing training of TBAs. Third, this pilot was conducted among a homogenous ethnic group in one health district which could limit generalisation to other regions of Guatemala and LMIC settings. Fourth, while we hypothesise that OCNs improved patient experience, we did not directly measure patient satisfaction or the actual incidence of disrespectful or abusive facility care. These would be a valuable component of future efforts.

As the first ever pilot of OCNs as a solution to mend the obstetric continuum of care, there are many questions yet to be answered. At the time of publication, our OCN intervention continues to assist referrals for the same group of TBAs. We hope to conduct a randomised trial of the OCN model powered to detect differences in maternal and neonatal outcomes alongside a rigorous assessment of patient satisfaction. If proven effective, as suggested by the improvement data presented here, the OCN model would offer a tremendous opportunity to test variants of programme design across different settings. For example, implementing the intervention in diverse health districts would help understand how public hospital leadership impacts intervention success. Similarly, a larger study could compare delivery of OCN services through TBAs as reported here to their formal integration into Ministry of Health centres or hybrid designs with birthing homes. Replication of the model in other cultural contexts could help define what programme components are essential to facilitate adaptation and scale-up.

## References

[1] Gruskin S, Cottingham J, Hilber AM, et al Using human rights to improve maternal and neonatal health: history, connections and a proposed practical approach. Bull World Health Organ 2008;86:589–93. 10.2471/blt.07.050500 18797615 PMC2649451

[2] World Health Organization (WHO), UNICEF, UNFPA Trends in maternal mortality: 1990 to 2015 - Estimates by WHO, UNICEF, UNFPA, World Bank Group and the United Nations Population Division 2015.

[3] World Health Organization (WHO) Maternal mortality Fact sheet #348, 2015 Available: http://www.who.int/mediacentre/factsheets/fs348/en/ [Accessed 12 Aug 2018].

[4] Hug L, Sharrow D, Zhong K, et al Levels & Trends in Child Mortality. United Nations Child Fund 2018.

[5] Alvarez JL, Gil R, Hernández V, et al Factors associated with maternal mortality in sub-Saharan Africa: an ecological study. BMC Public Health 2009;9:1–8. 10.1186/1471-2458-9-462 20003411 PMC2801510

[6] Brockerhoff M, Hewett P Inequality of child mortality among ethnic groups in sub-Saharan Africa. Bull World Health Organ 2000;78:30–41. 10686731 PMC2560588

[7] Secretaria de Planificacion y Programacion de la Presidencia (SEGEPLAN), Ministerio de Salud Pública y Asistencia Social [MSPAS] 2007. Guatemala City, Guatemala Estudio Nacional de Mortalidad Materna; 2011.

[8] Hussein J, Kanguru L, Astin M, et al The effectiveness of emergency obstetric referral interventions in developing country settings: a systematic review. PLoS Med 2012;9:e1001264–12. 10.1371/journal.pmed.1001264 22807658 PMC3393680

[9] Lassi ZS, Haider BA, Bhutta ZA Community-Based intervention packages for reducing maternal and neonatal morbidity and mortality and improving neonatal outcomes. Cochrane Database Syst Rev 2010:CD007754 10.1002/14651858.CD007754.pub2 21069697

[10] World Health Organization (WHO), Human Reproduction Programme, UNICEF Strategies toward ending preventable maternal mortality (EPMM). Geneva, Switzerland; 2015.

[11] Chary A, Díaz AK, Henderson B, et al The changing role of Indigenous lay midwives in Guatemala: new frameworks for analysis. Midwifery 2013;29:852–8. 10.1016/j.midw.2012.08.011 23410502

[12] Maupin JN Remaking the Guatemalan midwife: health care reform and midwifery training programs in highland Guatemala. Med Anthropol 2008;27:353–82. 10.1080/01459740802427679 18958785

[13] Berry NS Kaqchikel midwives, home births, and emergency obstetric referrals in Guatemala: contextualizing the choice to stay at home. Soc Sci Med 2006;62:1958–69. 10.1016/j.socscimed.2005.09.005 16225975

[14] Lassi ZS, Das JK, Salam RA, et al Evidence from community level inputs to improve quality of care for maternal and newborn health: interventions and findings. Reprod Health 2014;11:S2 10.1186/1742-4755-11-S2-S2 PMC4160921 25209692

[15] Goldman N, Glei DA Evaluation of midwifery care: results from a survey in rural Guatemala. Soc Sci Med 2003;56:685–700. 10.1016/S0277-9536(02)00065-5 12560004

[16] Homer CSE, Friberg IK, Dias MAB, et al The projected effect of scaling up midwifery. The Lancet 2014;384:1146–57. 10.1016/S0140-6736(14)60790-X 24965814

[17] Martinez B, Ixen EC, Hall-Clifford R, et al mHealth intervention to improve the continuum of maternal and perinatal care in rural Guatemala: a pragmatic, randomized controlled feasibility trial. Reprod Health 2018;15:120 10.1186/s12978-018-0554-z 29973229 PMC6033207

[18] World Health Organization (WHO) Standards for improving quality of maternal and newborn care in health facilities. Geneva, Switzerland; 2016.

[19] Austad K, Chary A, Martinez B, et al Obstetric care navigation: a new approach to promote respectful maternity care and overcome barriers to safe motherhood. Reprod Health 2017;14:148 10.1186/s12978-017-0410-6 29132431 PMC5683321

[20] Freeman HP The origin, evolution, and principles of patient navigation. Cancer Epidemiol Biomarkers Prev 2012;21:1614–7. 10.1158/1055-9965.EPI-12-0982 23045534

[21] Das JK, Kumar R, Salam RA, et al Evidence from facility level inputs to improve quality of care for maternal and newborn health: interventions and findings. Reprod Health 2014;11:S4–15. 10.1186/1742-4755-11-S2-S4 PMC4160922 25208539

[22] Bohren MA, Hofmeyr GJ, Sakala C, et al Continuous support for women during childbirth. Cochrane Database Syst Rev;2014 10.1002/14651858.CD003766.pub6 PMC6483123 28681500

[23] Stroux L, Martinez B, Coyote Ixen E, et al An mHealth monitoring system for traditional birth attendant-led antenatal risk assessment in rural Guatemala. J Med Eng Technol 2016;40:356–71. 10.1080/03091902.2016.1223196 27696915 PMC5180361

[24] Thaddeus S, Maine D Too far to walk: maternal mortality in context. Soc Sci Med 1994;38:1091–110. 10.1016/0277-9536(94)90226-7 8042057

[25] Leatherman S, Ferris TG, Berwick D, et al The role of quality improvement in strengthening health systems in developing countries. Int J Qual Health Care 2010;22:237–43. 10.1093/intqhc/mzq028 20543209

[26] Provost LP, Murray S The health care data guide: learning from data for improvement. San Francisco, CA: John Wiley & Sons, 2011.

[27] Benneyan JC Use and interpretation of statistical quality control charts. Int J Qual Health Care 1998;10:69–73. 10.1093/intqhc/10.1.69 10030790

[28] Linden A Conducting interrupted time-series analysis for single- and Multiple-group comparisons. Stata J 2015;15:480–500. 10.1177/1536867X1501500208

[29] Baum C, Schaffer M ACTEST: Stata module to perform Cumby-Huizinga General test for autocorrelation in time series. Stat Softw Components 2013.

[30] Pasha O, McClure EM, Wright LL, et al A combined community- and facility-based approach to improve pregnancy outcomes in low-resource settings: a global network cluster randomized trial. BMC Med 2013;11:1 10.1186/1741-7015-11-215 24090370 PMC3853358

[31] Franklin HL, Mirza W, Swanson DL, et al Factors influencing referrals for ultrasound-diagnosed complications during prenatal care in five low and middle income countries. Reprod Health 2018;15:1–9. 10.1186/s12978-018-0647-8 30541560 PMC6291965

[32] Yeoh Z-Y, Jaganathan M, Rajaram N, et al Feasibility of patient navigation to improve breast cancer care in Malaysia. J Glob Oncol 2018;4:1–13. 10.1200/JGO.17.00229 PMC7010457 30398950

[33] Bauserman M, Lokangaka A, Thorsten V, et al Risk factors for maternal death and trends in maternal mortality in low- and middle-income countries: a prospective longitudinal cohort analysis. Reprod Health 2015;12 Suppl 2:S5 10.1186/1742-4755-12-S2-S5 PMC4464034 26062992

[34] Afulani PA, Phillips B, Aborigo RA, et al Person-Centred maternity care in low-income and middle-income countries: analysis of data from Kenya, Ghana, and India. Lancet Glob Health 2019;7:e96–109. 10.1016/S2214-109X(18)30403-0 30554766 PMC6293963

[35] Peca E, Sandberg J Modeling the relationship between women’s perceptions and future intention to use institutional maternity care in the Western Highlands of Guatemala. Reprod Health 2018;15:1–17. 10.1186/s12978-017-0448-5 29325572 PMC5765672

[36] Downe S, Lawrie TA, Finlayson K, et al Effectiveness of respectful care policies for women using routine intrapartum services: a systematic review. Reprod Health 2018;15 10.1186/s12978-018-0466-y PMC5801845 29409519

