# Supplementary figures and images for: Obstetric care navigation: results of a quality improvement project to provide accompaniment to women for facility-based maternity care in rural Guatemala
Source: BMJ Qual Saf. 2019 Nov 2;29(2):169–78. doi: 10.1136/bmjqs-2019-009524 (PMC7045784; doi:10.1136/bmjqs-2019-009524)

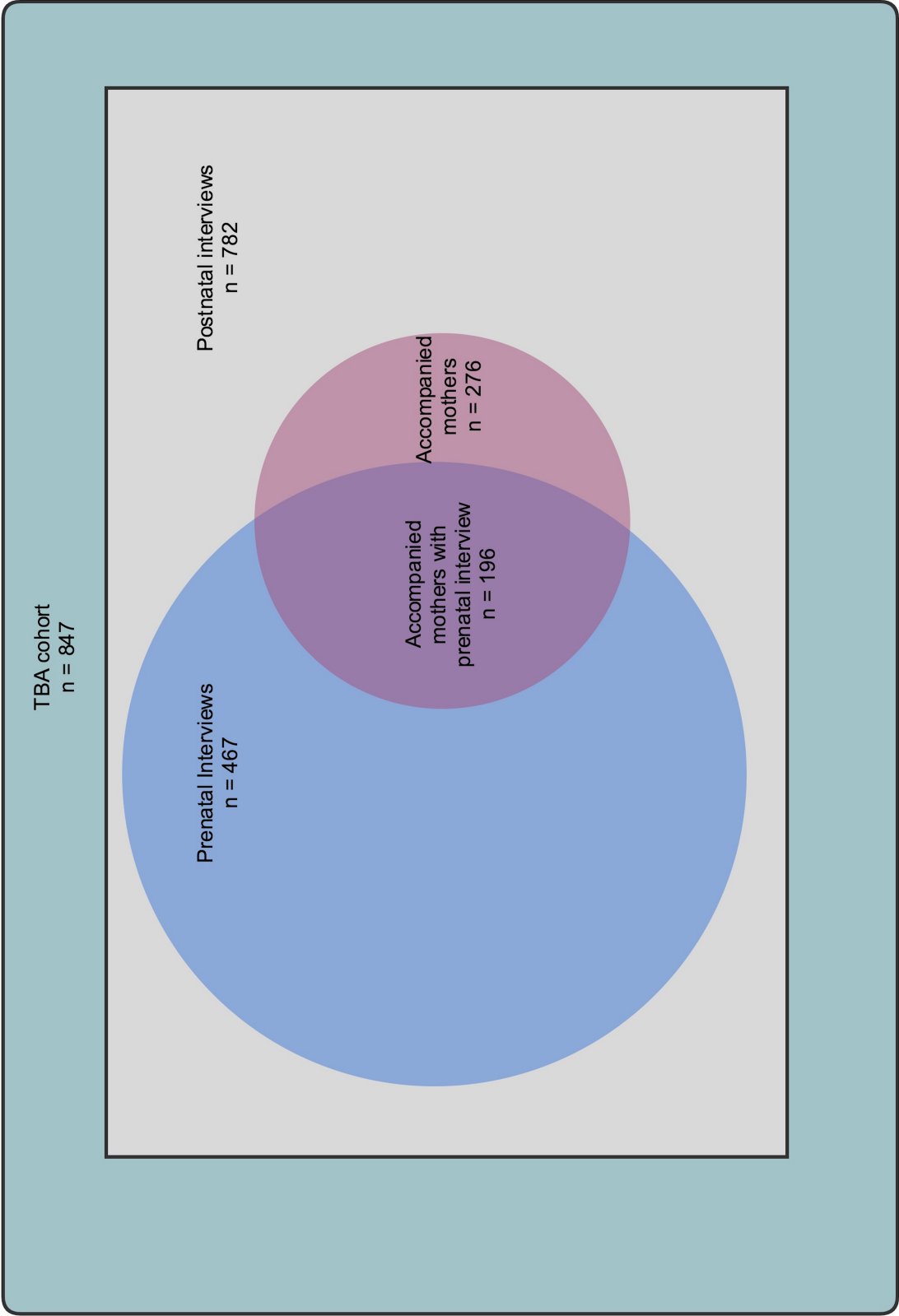

Supplement: Supplementary data [file bmjqs-2019-009524supp001.pdf]

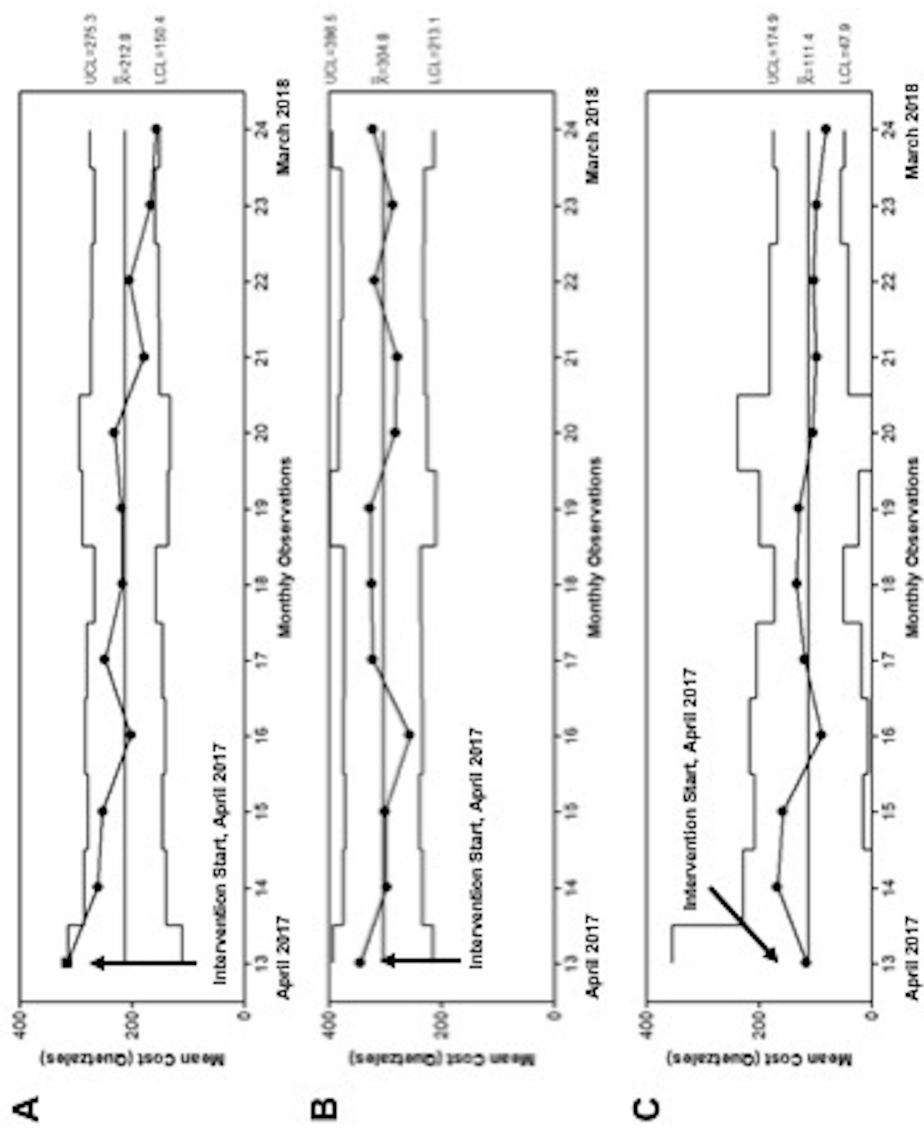

Supplement: Supplementary data [file bmjqs-2019-009524supp002.pdf]

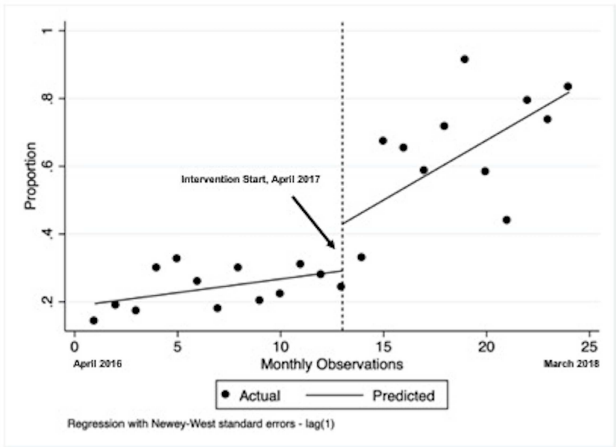

Supplement: Supplementary data [file bmjqs-2019-009524supp003.pdf]
